# Study on the Prosthesis Structural Design and Vibration Characteristics Based on the Conduction Effect of Human Middle Ear

**DOI:** 10.1155/2020/4250265

**Published:** 2020-05-20

**Authors:** Wu Ren, Huijuan Yan, Yi Yu, Jinghong Ren, Jinlong Chang, Yidong Wang, Yibo Han

**Affiliations:** ^1^School of Medical Engineering, Xinxiang Engineering Technology Research Center of Intelligent Rehabilitation Equipment, Xinxiang Neural Sensing and Control Engineering Research Center, Xinxiang Medical University, Xinxiang, Henan 453003, China; ^2^School of Pharmacy, Xinxiang Medical University, Xinxiang, Henan 453003, China; ^3^Huanghe Jiaotong University, Wuzhi, Henan 454950, China

## Abstract

As a bridge from the sound signal in the air to the sound perception of the inner ear auditory receptor, the tympanic membrane and ossicular chain of the middle ear transform the sound signal in the outer ear through two gas-solid and solid-liquid conversions. In addition, through the lever principle formed by three auditory ossicle structure, the sound was concentrated and amplified to the inner ear. However, the sound transmission function of the middle ear will be decreased by disease, genetic, or trauma. Hence, using middle ear prosthesis to replace the damaged ossicles can restore the conduction function. The function realization of middle ear prosthesis depends on the vibration response of the prosthesis from the tympanic membrane to the stapes plate on the human auditory perception frequency, which is affected by the way the prosthesis combined with the tympanic membrane, the material, and the geometric shape. In this study, reasonable prosthetic structures had been designed for different types of ossicular chain injuries, and the frequency response characteristics were analyzed by the finite element method then. Moreover, in order to achieve better vibration frequency response, a ball structure was designed in the prosthesis to simulate its amplification function. The results showed that the middle ear prostheses constructed by different injury types can effectively transfer vibration energy. In particular, the first- and second-order resonant frequencies and response amplitudes are close to each other when ball structure models of different materials are added. Instead, the resonance frequency of the third stage formed by aluminum alloy ball materials is larger than that of the other two, which showed good response features.

## 1. Introduction

Hearing as a communication channel plays an important role in human social life. Firstly, the vibrations of sound waves are collected by the auricle and travel from the external auditory canal to the eardrum, which causes mechanical vibrations between the eardrum and the ossicular chain. Secondly, the vibration signals are transmitted to the stapes plate, with the help of vestibular window, travel to the external lymph of the inner ear. Therefore, hearing comes into being. Thirdly, an ossicular chain is formed by malleus, incus, and stapes connected by joints, which is used to transmit sound waves. Moreover, the ossicular chain is an important sound transmission structure to maintain normal hearing. In particular, the damage of the three ossicles will result in impaired or interrupted sound transmission. However, when the ossicular chain is severely damaged, a replacement is needed to restore its conduction function to help restore hearing.

Wullstein first proposed the concept of tympanoplasty in the 1950s. He argued that the technique of ossicular chain reconstruction gradually matured and the therapeutic effect was remarkable with times going by. Many researchers have used partial or total auditory prosthesis for the reconstruction of the auditory chain while some researchers have studied the replacement of the auditory prosthesis. In 2015, Katilmis [1] et al., by analyzing the relationship between the success rate of transplantation and the degree of hearing improvement in patients with auditory ossicle replacement, concluded that the incidence of complications caused by the success rate of auditory ossicle replacement prosthesis was not large. Gentil et al. [2] used finite element to analyze the dynamic behavior of the middle ear in the whole auditory ostium replacement prosthesis and summarized that the use of cartilage with a diameter of 4 mm and thickness of 0.3 mm has had better repair effects. Piao et al. [3] established a three-dimensional model through computed tomography of the normal right ear, calculated the acoustic characteristics of the finite element, studied the influence of biofilm, and proposed that the growth of biofilm on the titanium ossicle would affect the recovery of hearing.

Gottlieb et al. [4] in 2016 analyzed the transfer function of stapes three-dimensional velocity and verified the conclusion that it had no interference to acoustic performance propagation. Gostian et al. [5] studied a number of cases of hearing recovery surgery and concluded that partial replacement of auditory ossicles had a good effect on hearing recovery, and its unique structural design could be placed stably without damage to other tissues. Gostian et al. [6] claimed that a new method for precentral perforation of the prosthesis was to make it safer to replace the prosthesis with the whole ostia. Kaftan [7] mentioned that three-dimensional reconstruction of stapes prosthesis through computed tomography proves that this method was feasible. Haifeng et al. [8] established and verified the validity of the whole ear model, used finite element to calculate its dynamic process, and studied its acoustic characteristics. Shaoxing and Furong [9] analyzed and summarized various research methods on the mechanics of the middle ear and proposed further research directions. In 2017, Rusineka et al. [10] declared that a shape memory alloy prosthesis was suitable for different patients by using cochlear implant to reconstruct the middle ear model. In order to solve the particularity of prosthesis required by patients, Hirsch et al. [11] advocated a solution of customized 3D printing of the personalized auditory bone, which effectively improved the success rate of reconstruction of auditory bone chain. Kamrava and Roehm [12] analyzed the difference of each dimension in auditory skeleton, customized accessories and optimized the fitting degree of prosthesis based on a given variation range, and then modified the model according to the specificity.

Lahlou et al. [13] in 2018 analyzed the way in which the titanium alloy ostium chain was used to replace prosthesis in surgical patients and argued that ostium formation with titanium prosthesis was conducive to hearing rehabilitation. Saliba [14] analyzed the results of the patients' surgical conditions by using the two methods and believed that the new fat prosthesis was more applicable than the method of total hearing small bone replacement prosthesis.

In 2019, Haidar et al. [15] tracked the postoperative situation of patients, studied the effect of cervical vertebra space resection under endoscope on auditory ostium formation, and presented that it could reduce the surgical risk and improve the function of this part.

To sum up, the existing research has been studied and applied on the auditory ossicular replacement surgery and postoperative hearing recovery, and the ossicular chain has been mostly simplified as a bar in clinical application. On the other hand, with the stimulation of low frequency, the vibration mode of the ossicular chain in the stapes foot plate was characterized by piston motion while under high frequency, the ability of the stirrup plate to swing was low. So, its frequency response characteristics were not good under a high frequency and the hearing conduction was also affected. The quantitative research on the design and vibration characteristics of different middle ear hearing prostheses is not enough, and the research on the swing motion of the stapes plate and comprehensive comparison on the replacement of prostheses in various parts is insufficient. Accordingly, this paper will help the patients with impaired ossicular chain restore their basic hearing. By improving the design of ossicular prosthesis and optimizing the single model, the prosthesis can be more in line with the motion mode of the ossicular bone. Furthermore, studying the vibration characteristics of different prostheses, the characteristics of different prostheses will be obtained. In general, six replacement methods including the tympanic membrane contact site, malleus, stapes, incus, partial ossicular chain, and full ossicular prosthesis will be included in this study; in the meantime, the clinical methods of hearing reconstruction in the middle ear will be summarized. More than ever, the elastic ball adopted in the replacement of full ostium prosthesis will better solve the vibration conduction of stapes plate at a high frequency, provide a new clinical treatment method, and make a reference for the design of other similar medical devices.

## 2. The Modeling of Prosthesis

**Figure 1:**
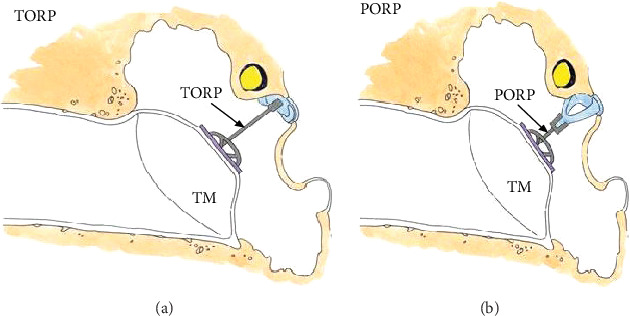
Schematic diagram of the middle ear. (a) The prosthetic replacement for the lack of middle ear stapes and incus. (b) The prosthetic replacement for the lack of middle ear incus.

Different prosthetic replacement schemes were used for different types of ossicular chain injuries. In addition, from the anatomical feature, the design of ossicular prosthesis must be based on the anatomic structure of the tympanic chamber. The shape or size of the prostheses is the basis of the reconstruction of the middle ear. The modeling process was as follows. Firstly, CT scan images of normal and injured middle ear ossicular chains were obtained from the hospital, and the sizes of ossicular prostheses were obtained by comparing normal and injured ones. After which, three-dimensional prosthesis models of malleus, stapes, incus, all ossicular chains, and tympanic membrane contact sites were established by Creo Parametric 3D Modeling Software. Secondly, in Ansys software, 0.632 Pa (90 dB) uniform sound was applied to the finite element model of the component to conduct harmonic response analysis with the frequency range from 200 to 8000 Hz. Meanwhile, the effectiveness and reliability analysis of the component were obtained. Then, the size and shape of the prosthesis have been improved till the reasonable size was obtained. The prosthetic replacements for different types of the middle ear are shown in Figure 1.

### 2.1. The Top Plate of the Prosthesis

**Figure 2:**
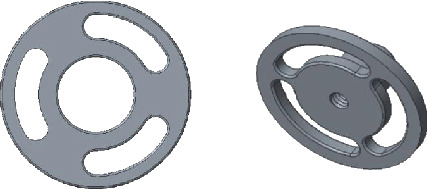
The top plate of the prosthesis.

The top plate mostly is made into a round or oval shape and can also be made into a “T” or an “L” shape to prevent it from contacting ossification and adhesion with the back wall of tympanum. The design is shown in Figure 2.

### 2.2. The Handle Design of the Prosthesis

**Figure 3:**
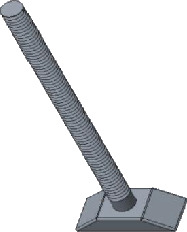
The handle of the prosthesis.

The diameter of the prosthetic handle depends on the material elasticity and size, which is effectively appropriate to support the prosthetic, conduct sound waves, and increase the safety of the operation. The length of the handle should be slightly lifted up the tympanic membrane. Otherwise, too long can compress the tympanic membrane and cause bedsore necrosis, while too short can lead to conductive hearing loss. The design is shown in Figure 3.

### 2.3. The Connected Ball

**Figure 4:**
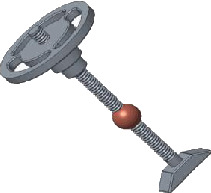
The ball and its location.

In the case of high-frequency stimulation, the swing ability of the stapes plate of the prosthesis is low and the frequency response characteristic is not good. However, the ball in the connecting handle (shown in Figure 4) does not have an effect on the low-frequency stimulation. The piston motion of the stapes foot plate can reduce the rotation effect of the prosthesis handle of the anterior and posterior axis under high-frequency stimulation; thus, the frequency response characteristics are optimized.

### 2.4. The Bottom Design of the Prosthesis

**Figure 5:**
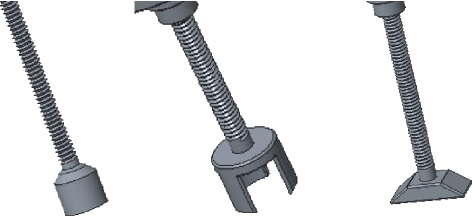
Different bottom connections of the prosthesis.

According to the degree of damage to the ossicular chain, the bottom connection forms of cylinder, clip, and flat base are, respectively, designed (shown in Figure 5), so as to meet the stability of the ossicular chain in different conditions.

### 2.5. Different Types of Ossicular Prosthesis

**Figure 6:**
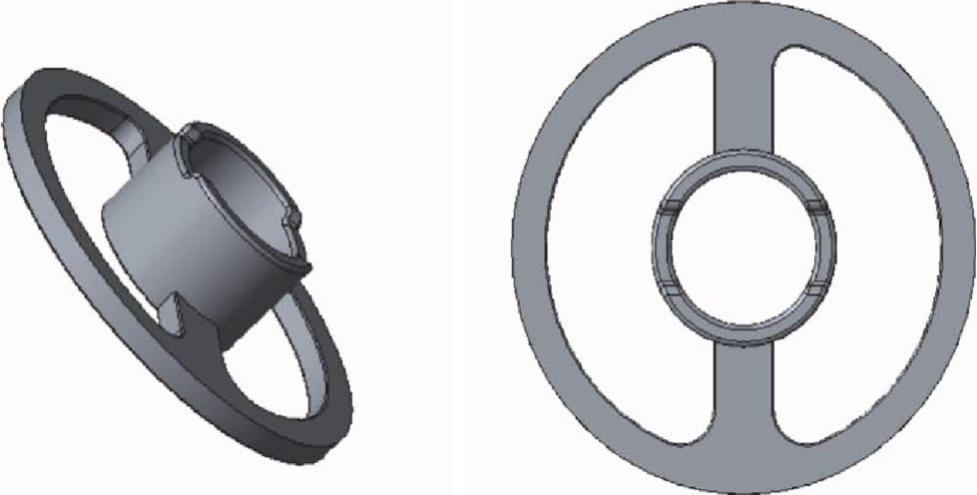
Simplified ossicular prosthesis.

A simplified ossicular prosthesis for the loss of ossicles at the eardrum contact site is shown in Figure 6.

**Figure 7:**
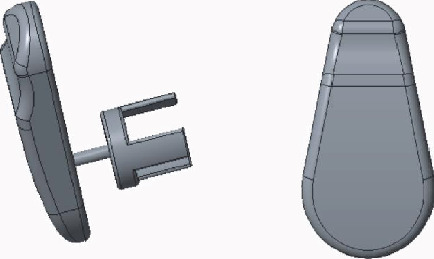
Modified ossicular prosthesis.

An improved prosthesis of the ossicles missing near the eardrum is shown in Figure 7. A small piece of titanium structure is used to connect the top plate with the shank, which can be bent at any angle to make the top plate move in the same direction as the eardrum.

**Figure 8:**
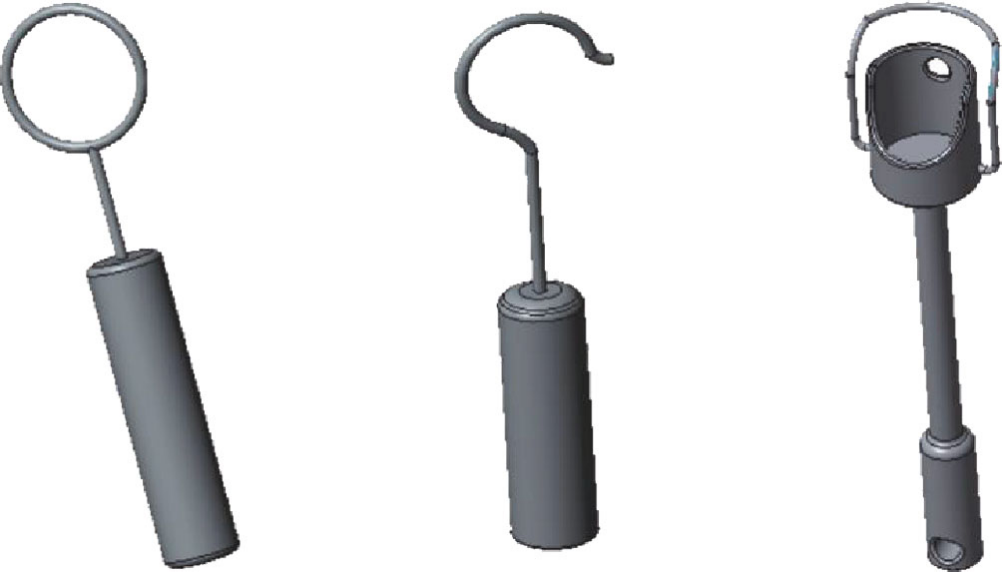
Different types of prosthesis for stapes injury.

The stapedectomy prosthesis is shown in Figure 8.

**Figure 9:**
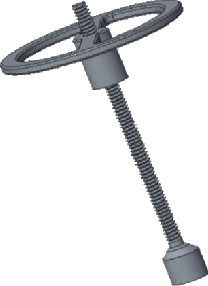
Stapedectomy with incus prosthesis.

The stapedectomy with missing incus prosthesis is shown in Figure 9.

**Figure 10:**
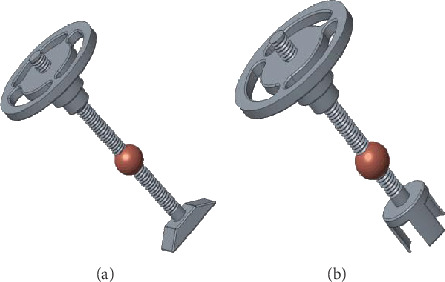
(a) All missed ossicular chain model. (b) Partial missed ossicular chain model.

When the ossicular chain is partially damaged, the lower end of the handle is made into a hollow shape or cut into a petal shape, which is convenient to cover and fix in the head of stapes. When the ossicular chain is completely missing, the end of the handle is cupped and placed in the stapes microcephalus which enhances the stability. In order to increase the hearing aid effect at a high frequency, an elastic ball scheme in the middle of the connecting handle of the prosthetic has been proposed. The designed prosthetic objects are shown in Figure 10.

## 3. The Finite Element Analysis of Prosthesis

In order to study the vibration characteristics of different prosthesis, the harmonic response analysis had been carried out in Ansys software. Creo Parametric 3D Modeling Software had been used to establish the three-dimensional models. The first type is a pure titanium alloy model, the second is the model with aluminum alloy ball in the middle, and the third is the prosthesis with the copper alloy ball in the handle. Before importing the prosthesis into Ansys/workbrench, the CAE model was carried out to remove the features that had little influence on the overall result but occupied the analysis resources and time, to suppress the small chamfer and hole that avoid the appearance of the phenomena that the analysis stress concentrates, calculation stops, and crashes. After that, Ansys was imported to establish the finite element analysis model. The pressure has been applied on the top surface of the prosthesis cylinder, and the boundary constraints located on the prosthesis top cylinder side. The element type is defined as solid 187 (10-node, tetrahedral elements). Besides the titanium alloy material density is 4620 kg/m^3^, elastic modulus is 9.6*E*+10 Pa, Poisson's ratio is 0.34. The aluminum alloy density is 2270 kg/m^3^, elastic modulus is 7.1*E*+10 Pa, Poisson's ratio is 0.33. And the copper alloy is density is 8300 kg/m^3^, elastic modulus is 1.1*E*+11 Pa, Poisson's ratio is 0.326. Furthermore, the node number of the prosthesis model of pure titanium alloy is 21336, and the element number is 12547; besides, the node number of the prosthesis model including aluminum alloy or copper alloy ball is 24065 and the element number is 14613. Among the 14613 elements, the ball node number is 458, and the element number is 224. After the constraints of displacement and load are established, the harmonic responses at different frequencies are analyzed.

## 4. The Discussion on Vibration Characteristics of Different Prosthesis Types

In order to study the vibration characteristics of the prosthesis under high-frequency vibration, a frequency of 5562.5 Hz is selected for harmonic response analysis, and the results are as follows.

**Figure 11:**
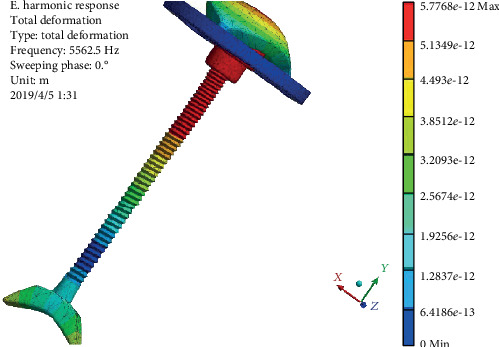
Displacement cloud of prosthesis pure titanium alloy.

The vibration displacement diagram of the pure titanium alloy prosthesis harmonic response model is shown in Figure 11. It can be seen that the maximum displacement at the frequency of 5562.5 Hz reaches 5.78 × 10^−9^ mm, which occurs at the top of the prosthesis and the top of connecting handle. That is, at the connection between the external ear and the prosthesis, the vibration displacement reaches the maximum, then increases again at the connection between the top plate and the connecting handle, and then decreases gradually at the bottom of the connecting handle.

**Figure 12:**
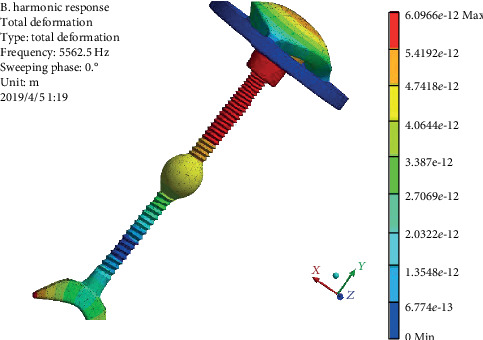
Displacement cloud diagram of the prosthesis with aluminum alloy ball.

In order to increase the vibration amplitude and expand the energy transfer effect, the aluminum alloy ball is added into the second design. The harmonic response results are shown in Figure 12. It can be seen that the maximum vibration response displacement of the component reaches 6.09 × 10^−9^ mm after the addition of aluminum alloy ball, which indicates that the addition of aluminum alloy ball increases the vibration amplitude and the sound transmission effect. Its displacement response rule is similar to that of pure titanium alloy connecting handle; both of which have a large displacement from the top of the connection of the outer ear, which gradually decreases to the top of the handle, then increases at the bottom of the connection of the top plate, and then gradually decreases to the base. Compared with pure titanium alloy handle, the vibration amplitude after adding the aluminum alloy ball is larger in the upper part of the connecting handle and the displacement is about 4.0 × 10^−9^ mm. When it comes to the bottom of the connecting handle, the vibration displacement gradually decreases.

**Figure 13:**
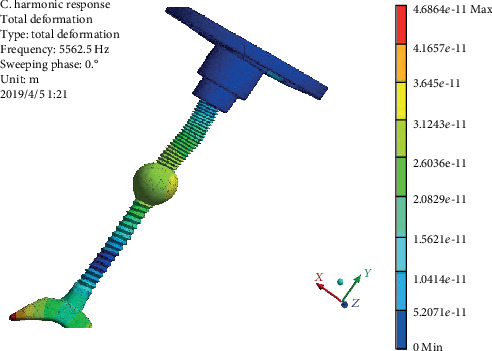
Displacement cloud diagram of prosthesis with the copper alloy ball.

To verify the influence of different connection ball vibration displacements, the copper alloy ball has been used to replace the aluminum alloy ball to study different material influence on the energy transfer and harmonious response of the structure. It is observed that the maximum vibration displacement of the prosthesis with the copper alloy ball is about 4.7 × 10^−9^ mm in Figure 13; moreover, the change rule is similar to the above two models.

**Figure 14:**
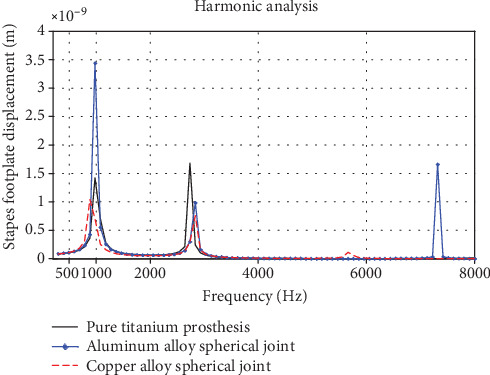
Three different structural types of prosthesis-complex harmonic response analysis curves.

The analysis and comparison of three different prosthesis harmonic responses are shown in Figure 14. From the above discussions, we can predict that the maximum displacement of vibration amplitude of three different prosthesis harmonic responses between the frequency 800 Hz and 2300 Hz has little difference. When the frequency is larger than 5000 Hz, the vibration amplitude of the prosthesis added with the alloy ball did not decrease too much; especially, the displacement effect of the aluminum alloy's vibration harmonic response is obvious near 7000 Hz. As can be seen from the comparison, the prosthesis with the aluminum alloy ball is more widely used according to the frequency response curve and has a better frequency adaptation stage, which has a better effect than that of the copper alloy ball. And the vibration transmission effect of pure titanium alloy prosthesis in the high frequency has a relatively poor effect.

## 5. Conclusion

We can infer that the applicability of pure prosthesis between different frequency ranges is not as good as that of that with the added ball alloy component. Personalized design was carried out, and the most suitable ossicular prosthesis can be developed according to the injury conditions of different patients, which will reduce the probability secondary injury. Firstly, the top plate of the ossicular prosthesis was designed with a small groove to facilitate the attachment of cells on the surface of the tympanic membrane and enhance the fastness of the front-end connection. Secondly, a good conduction material was used to fabricate the prosthetic handle to increase the conduction effect of the prosthesis. Thirdly, the firmness of the connection between the end of the prosthesis and the stapes were increased by clamping and sticking. Fourthly, the elastic ball was adopted to reflect the vibration mode of the stapes plate and improve the conduction ability of the prosthesis under a high frequency.

Finally, the patient of the hearing impairment caused by different degrees of middle ear injury was analyzed in this study, the model of middle ear prosthesis was designed according to different conditions, and a new type of ossicular prosthesis has been designed. Besides, by optimizing the structure, material, and connection mode, the current market product with a single-link mode has been changed. The use of the connected ball made the motion mode of the ossicular prosthesis and stapes plate more consistent with the actual situation, and the stability of the prosthesis has been enhanced. With the help of harmonic response analysis of the three different prosthesis models, it can be concluded that the prosthesis with the aluminum alloy ball has had the best application effect and the widest application range, which has provided data help for the promotion of clinical rehabilitation.

## Data Availability

The data used to support the findings of this study are available from the corresponding author upon request.
